# Multi-Omic Integration of the miRNome and Transcriptome in Dermal Fibroblasts from Hypermobile Ehlers–Danlos Syndrome and Hypermobility Spectrum Disorders Identifies Convergent Candidate miRNA-mRNA Regulatory Networks Linking Extracellular Matrix Remodeling and Inflammation

**DOI:** 10.3390/ijms27198628

**Published:** 2026-09-26

**Authors:** Iulia Andreea Pelisenco, Valeria Cinquina, Nicola Chiarelli, Valeria Bertini, Paolo Martini, Giulia Carini, Raffaele Mazzoleni, Cintia Castiglioni, Marian Viola, Jelena Skripac, Nicoletta Zoppi, Marina Colombi, Marco Ritelli

**Affiliations:** 1 Department of Molecular and Translational Medicine, Università degli Studi di Brescia, 25123 Brescia, Italy; pelisenco.iulia@gmail.com (I.A.P.); valeria.cinquina1@unibs.it (V.C.); nicola.chiarelli@asst-spedalicivili.it (N.C.); valeria.bertini@unibs.it (V.B.); paolo.martini@unibs.it (P.M.); giulia.carini@unibs.it (G.C.); r.mazzoleni002@studenti.unibs.it (R.M.); cintia.castiglioni@unibs.it (C.C.); marian.viola@unibs.it (M.V.); jelena.skripac@unibs.it (J.S.); nicoletta.zoppi@unibs.it (N.Z.); marina.colombi@unibs.it (M.C.); 2 Laboratory of Histopathology and Immunohistochemistry, Victor Babeș National Institute of Pathology, 050096 Bucharest, Romania; 3 Medical Genetics Laboratory, ASST Spedali Civili of Brescia, 25123 Brescia, Italy

**Keywords:** hypermobile Ehlers–Danlos syndrome, hypermobility spectrum disorders, microRNA, miRNome, extracellular matrix, fibroblast, inflammation, multi-omics

## Abstract

Hypermobile Ehlers–Danlos syndrome (hEDS) and hypermobility spectrum disorders (HSD) are clinically defined connective tissue conditions lacking molecular biomarkers. We tested whether their transcriptomic convergence extends to miRNA-mediated post-transcriptional regulation. miRNome sequencing was performed in cultured dermal fibroblasts from 12 individuals with hEDS, 12 with HSD, and 24 controls. Twenty-five differentially expressed miRNAs (DE-miRNAs) were assessed by qPCR in pooled discovery material and an independent pooled cohort. No miRNA met differential-expression threshold in the direct hEDS versus HSD comparison, whereas pooled hEDS/HSD analysis identified 88 DE-miRNAs versus controls (42 upregulated, 46 downregulated). qPCR supported cross-platform and pooled group-level concordance but did not assess interindividual validation. Experimentally supported targets of upregulated DE-miRNAs overlapped 388 downregulated genes significantly more than expected by chance; these genes converged on extracellular matrix remodeling, TGF-β/BMP, Wnt/Hippo, focal adhesion, and cytoskeletal programs. The 27-gene inverse overlap for downregulated DE-miRNAs did not exceed chance expectation but included literature-supported chemokine and matrix-degradative candidates. These hypothesis-generating findings define a shared group-level fibroblast miRNA pattern and prioritize candidate regulatory axes. Because the study used cultured cells and pooled qPCR without paired biofluids, longitudinal sampling, or phenotype correlations, its in vivo and biomarker implications remain preliminary.

## 1. Introduction

Hypermobile Ehlers–Danlos syndrome (hEDS) and hypermobility spectrum disorders (HSD) are clinically overlapping connective tissue conditions characterized by generalized joint hypermobility, chronic musculoskeletal pain, and variable multisystem manifestations that can markedly impair quality of life [1,2]. Unlike molecularly defined Ehlers–Danlos syndrome subtypes, hEDS and HSD have no established genetic cause or validated laboratory test and are diagnosed using the 2017 clinical framework [1,3]. Under this framework, hEDS requires generalized joint hypermobility, specified systemic features, and exclusion of alternative diagnoses, whereas symptomatic individuals who do not meet all hEDS criteria are classified within HSD [3]. Together, these conditions may affect more than 1 in 300–500 people [4,5].

The stringency of the 2017 criteria and the extensive phenotypic overlap have sustained debate over whether hEDS and HSD are biologically distinct disorders or different expressions of a shared continuum [6,7,8,9]. In a large clinical cohort, we found comparable patterns of extra-articular comorbidity and no clear clinical discontinuity at the current diagnostic boundary, supporting investigation of common molecular mechanisms while acknowledging substantial interindividual heterogeneity [8].

At the cellular level, hEDS and HSD dermal fibroblasts share extracellular matrix (ECM) disorganization and a myofibroblast-like phenotype driven by an αvβ3 integrin-ILK-Snail1/Slug axis; this phenotype can be induced in control fibroblasts and is partly reversed by matrix metalloproteinase (MMP) inhibition [10,11]. RNA sequencing subsequently showed no discriminating transcriptomic signature between hEDS and HSD, whereas the pooled patient group displayed coordinated downregulation of ECM organization, adhesion, migration, and cytoskeletal programs and upregulation of chemokine, leukocyte-activation, and inflammatory pathways [12]. These findings supported a model in which excessive ECM proteolysis and damage-associated signals reinforce a degradative-inflammatory loop that compromises connective-tissue homeostasis [12].

MicroRNAs (miRNAs) add a post-transcriptional regulatory layer to this model by promoting target messenger RNA (mRNA) degradation or translational repression and by coordinating multi-gene networks involved in ECM turnover, fibrosis, inflammation, and cell-state transitions [13,14,15,16,17]. Their dysregulation is increasingly recognized across hereditary and acquired connective-tissue disorders and may offer both mechanistic information and biomarker potential [18,19].

In osteogenesis imperfecta, fibroblast and circulating-serum miRNA profiles have been linked to COL1A1/COL1A2 defects and clinical severity, while peripheral-blood miRNA dysregulation has been reported in Marfan syndrome [20,21,22]. In vascular Ehlers–Danlos syndrome, integrated miRNome, transcriptome, and proteome analyses identified miR-29b-3p as a regulator of ECM and stress-response genes, with partial rescue after miR-29b inhibition in patient fibroblasts [23,24]. Acquired fibrosing connective-tissue diseases provide complementary evidence: plasma miRNA patterns stratify systemic-sclerosis subgroups, miR-196a is reduced in both involved skin and serum in localized scleroderma, and miR-181a/b modulates dermal fibrosis in vivo [25,26,27].

Other hereditary matrix disorders, including Loeys–Dietz syndrome and pseudoxanthoma elasticum, also converge on TGF-β/BMP signaling, cell–ECM dysfunction, myofibroblast activation, and MMP-dependent remodeling, although disease-specific miRNA data remain sparse [28,29,30,31]. We therefore profiled hEDS and HSD fibroblast miRNAs and integrated them with our transcriptome to prioritize candidate miRNA-mRNA axes for mechanistic and translational validation.

## 2. Results

### 2.1. Global Analysis of miRNA Expression Profiles in hEDS and HSD Dermal Fibroblasts

We performed miRNome sequencing on dermal fibroblast strains from 12 hEDS, 12 HSD, and 24 healthy control (CTRL) individuals selected from the cohort previously analyzed by RNA sequencing [12]. Use of matched cell strains and culture conditions enabled direct integration of the two omic layers. hEDS and HSD were initially analyzed separately to test whether their miRNA profiles were distinguishable.

**Figure 1:**
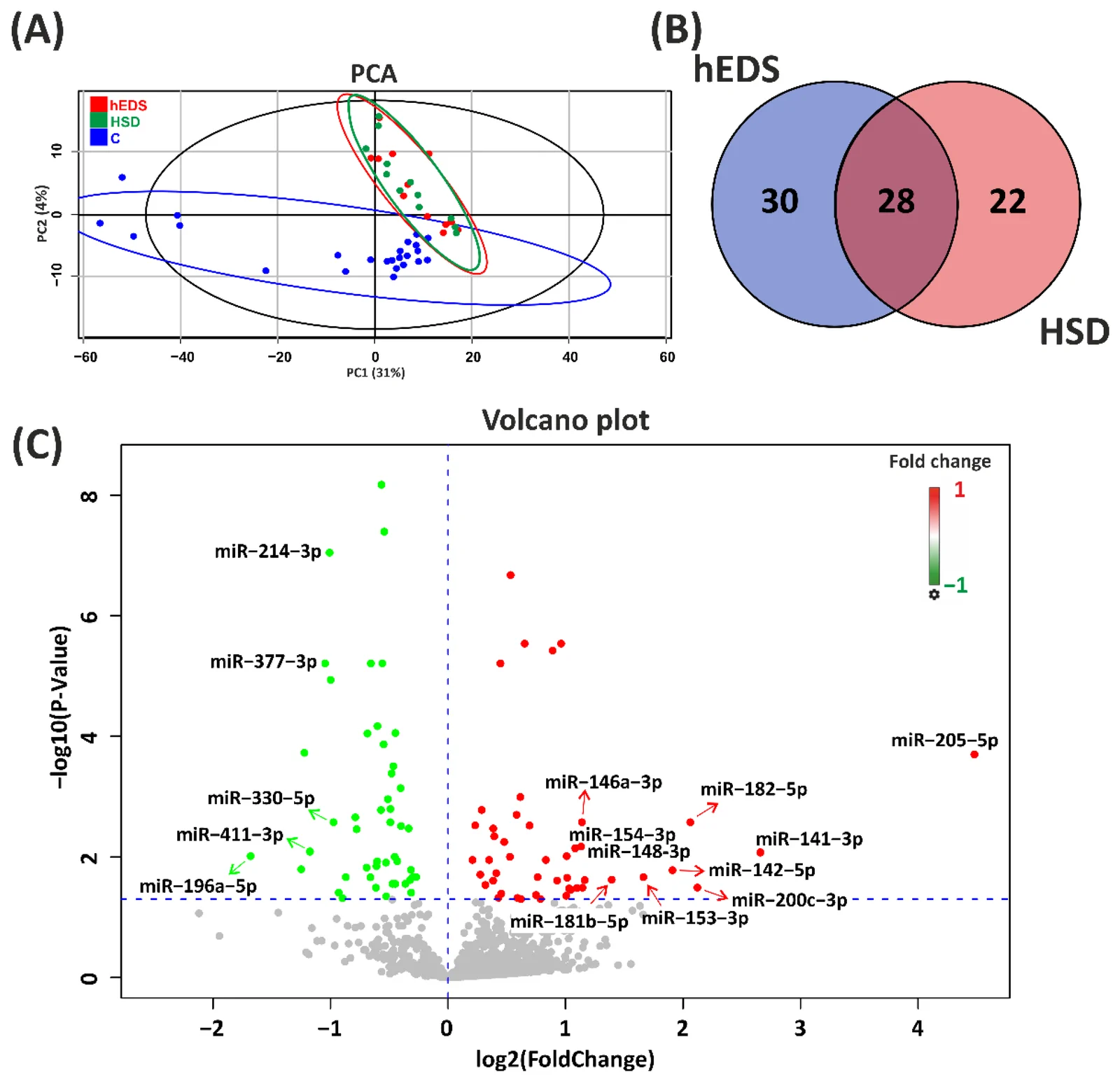
Global analysis of miRNA expression in dermal fibroblasts from hEDS, HSD, and CTRL subjects. (**A**) PCA plot displaying hEDS (red), HSD (green), and CTRL (blue) samples separately. hEDS and HSD occupy extensively overlapping regions, whereas most CTRL samples separate along PC1; PC1 and PC2 explain 31% and 4% of total variance, respectively. Ellipses are shown as visual guides to group dispersion. (**B**) Venn diagram of significant patient-versus-control comparisons: 28 DE-miRNAs overlap, while 30 occur only in the hEDS versus CTRL list and 22 only in the HSD versus CTRL list. These non-overlapping sets are comparison-specific and do not imply biological specificity for either diagnosis. (**C**) Volcano plot of the 88 DE-miRNAs in pooled hEDS/HSD fibroblasts versus CTRL, with 42 upregulated miRNAs in red and 46 downregulated miRNAs in green.

Separate comparisons identified 58 DE-miRNAs in hEDS versus CTRL and 50 in HSD versus CTRL, whereas no miRNA reached the adjusted *p*-value threshold of 0.05 in the direct hEDS versus HSD comparison (Table S1). In the three-group principal component analysis, hEDS and HSD samples occupied extensively overlapping regions, whereas most CTRL samples separated along PC1, which explained 31% of the total variance; PC2 explained 4% (Figure 1A). The two patient versus CTRL lists shared 28 significant miRNAs with concordant directionality; the remaining 30 hEDS-comparison and 22 HSD-comparison miRNAs generally changed in the same direction in both patient groups but reached significance in only one comparison (Figure 1B; Table S1). These non-overlapping entries should therefore be interpreted as comparison-specific rather than disease-specific.

The absence of statistically significant miRNAs in the direct comparison, together with the concordant patient versus CTRL trends and the previously observed transcriptomic convergence, provided a pragmatic basis for pooled downstream discovery analysis; it does not establish biological identity between hEDS and HSD. Comparison of the pooled hEDS/HSD group with CTRL identified 88 DE-miRNAs, including 42 upregulated and 46 downregulated species (Figure 1C; Table S1).

Because miRNome profiling was performed after ex vivo expansion, the observed differences should be interpreted within the standardized culture context. Culture duration, passage number, serum exposure, confluence, substrate mechanics, and other environmental variables can alter fibroblast state and miRNA abundance. All patient and control strains were processed in parallel using the same medium, a 72 h culture interval, approximately 80% confluence, and matched early passage numbers (passages 3–5), thereby limiting systematic technical differences between groups. Nevertheless, these controls cannot exclude culture adaptation or establish that the measured profiles directly reproduce the in vivo miRNome.

### 2.2. qPCR Evaluation of Selected DE-miRNAs

**Figure 2:**
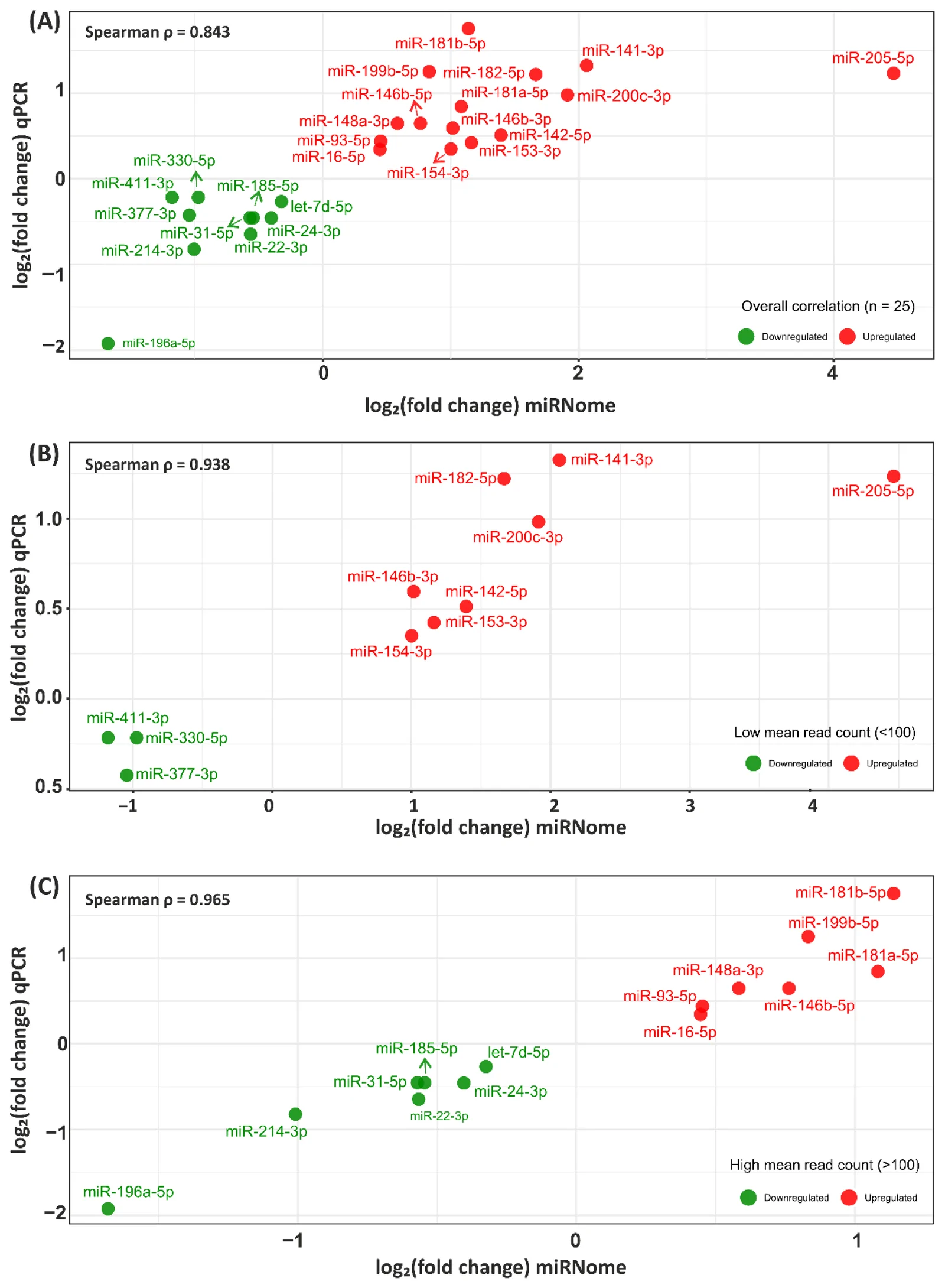
Correlation between miRNome and qPCR fold-change values for the 25 selected DE-miRNAs. (**A**) Overall relationship between log2(fold change) measured by miRNome sequencing and qPCR (Spearman’s ρ = 0.843). (**B**) Correlation for miRNAs with mean read count < 100 (ρ= 0.938). (**C**) Correlation for miRNAs with mean read count > 100 (ρ = 0.965). Upregulated miRNAs are shown in red and downregulated miRNAs in green. All correlations remained significant after multiple-testing correction (adjusted *p* < 0.001).

To assess platform concordance, qPCR was performed on pooled cDNA from the same fibroblast strains used for miRNome sequencing. Twenty-five DE-miRNAs were purposefully selected to represent both directions of change (15 upregulated and 10 downregulated) and to span the observed ranges of fold-change magnitude and mean read count, rather than restricting assessment to the most extreme or most abundant candidates (Table S2). miRNome and qPCR log2(fold-change) estimates showed strong rank concordance across the 25-miRNA set (Spearman’s ρ = 0.843, adjusted *p* < 0.001; Figure 2A), supporting agreement in direction and relative magnitude for the selected candidates.

Concordance remained strong after stratification by abundance: Spearman’s ρ was 0.938 for the 11 miRNAs with mean read count < 100 and 0.965 for the 14 miRNAs with mean read count > 100 (both adjusted *p* < 0.001; Figure 2B,C). Because these analyses used pooled material and group-level fold changes, they assess cross-platform agreement rather than interindividual reproducibility.

**Figure 3:**
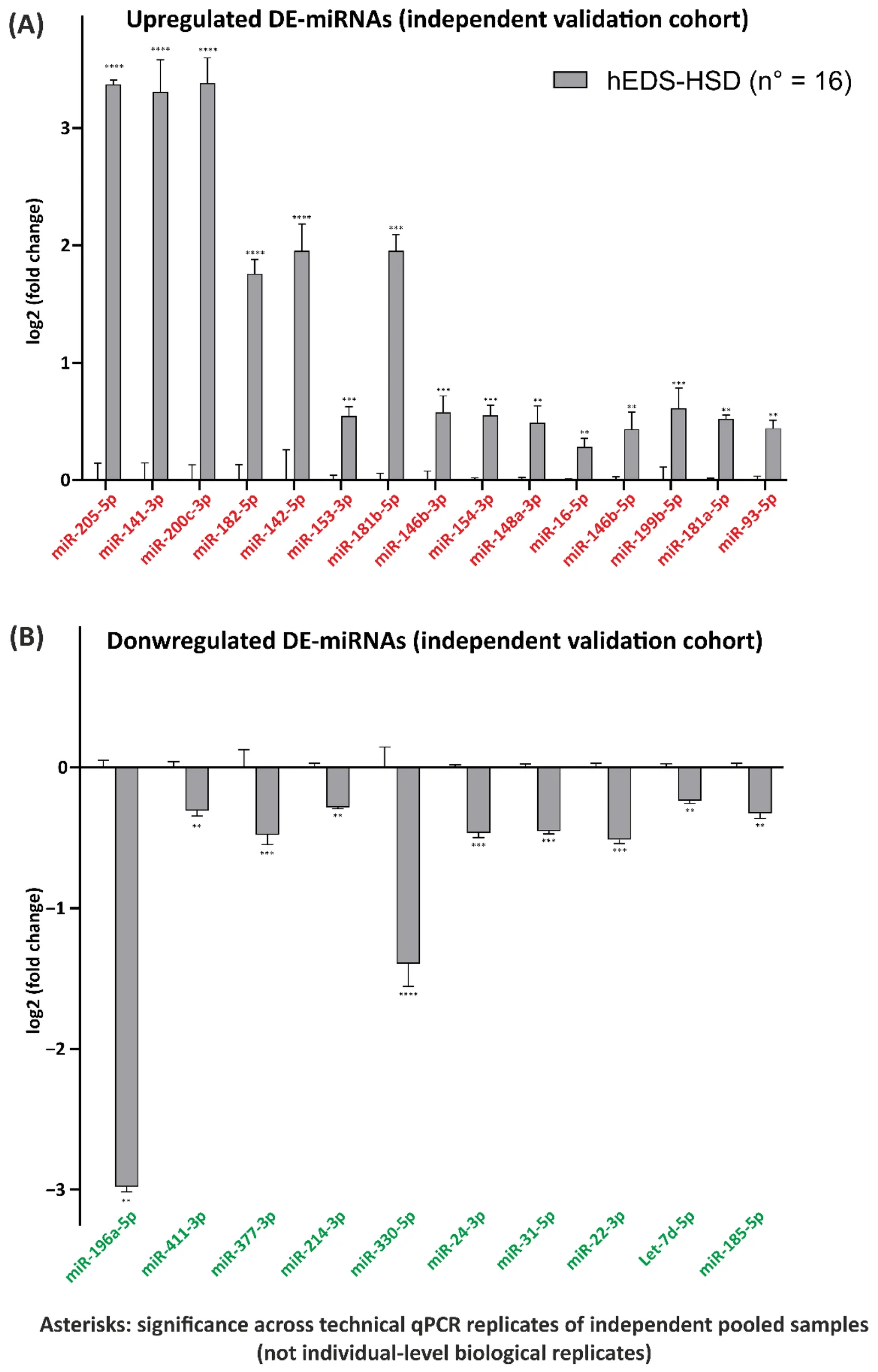
qPCR assessment of upregulated and downregulated DE-miRNAs in an independent pooled fibroblast cohort. (**A**) The 15 upregulated and (**B**) the 10 downregulated selected DE-miRNAs were measured in one pooled RNA sample generated from 8 hEDS, 8 HSD, and 16 CTRL subjects not included in miRNome sequencing. Bars show mean log2(fold change) ± SEM from repeated qPCR measurements relative to the CTRL pool, used as calibrator (fold change = 1; log2(fold change) = 0), calculated by the 2^−ΔΔCt^ method. Asterisks derive from one-sample tests across technical measurements of the same pooled biological material and do not indicate subject-level biological significance. ** *p* < 0.01, *** *p* < 0.001, **** *p* < 0.0001.

The same 25 miRNAs were then examined in pooled RNA from an independent set of 8 hEDS, 8 HSD, and 16 CTRL fibroblast strains drawn from the original RNA-seq cohort [12]. qPCR reproduced the direction of change for all 15 upregulated and 10 downregulated candidates (Figure 3). This experiment provides pooled group-level replication in an independent pooled cohort, but it cannot quantify between-subject variability or establish individual-level diagnostic performance.

### 2.3. Identification and Functional Characterization of Experimentally Supported Targets of the 88 DE-miRNAs

Experimentally supported targets of the 88 DE-miRNAs were retrieved with multiMiR by restricting the query to the validated components of miRTarBase, miRecords, and TarBase [32]. This excluded prediction-only interactions, but the retained evidence still ranged from direct reporter and protein assays to high-throughput binding or expression studies performed in diverse cellular contexts. The number of targets per miRNA therefore varied widely (median 150; IQR 80.75–309.25), reflecting both biology and uneven literature coverage (Table S3). After merging the 88 lists and removing duplicates, 9198 unique target genes were retained.

Enrichment analysis of this broad target universe identified gene-expression control, apoptosis, and cell migration among the leading biological processes; kinase- and transcription-related functions; and focal-adhesion and cell–substrate junction components (Figure S1). MAPK signaling, endocytosis, actin-cytoskeleton regulation, TGF-β, Wnt, and chemokine signaling were also enriched (Table S4). Given the size and literature bias of the 9198-gene universe, this analysis is best viewed as a pathway-level survey, with the directional integration described below providing greater disease specificity.

### 2.4. Integration of Up- and Downregulated miRNA Targets with hEDS/HSD DEGs

We next integrated the miRNome with the 952 DEGs previously identified in matched hEDS/HSD fibroblast strains [12]. Because miRNAs commonly reduce target-mRNA abundance or translation [13,33], experimentally supported targets of upregulated miRNAs were compared with downregulated DEGs, and targets of downregulated miRNAs were compared with upregulated DEGs.

**Figure 4:**
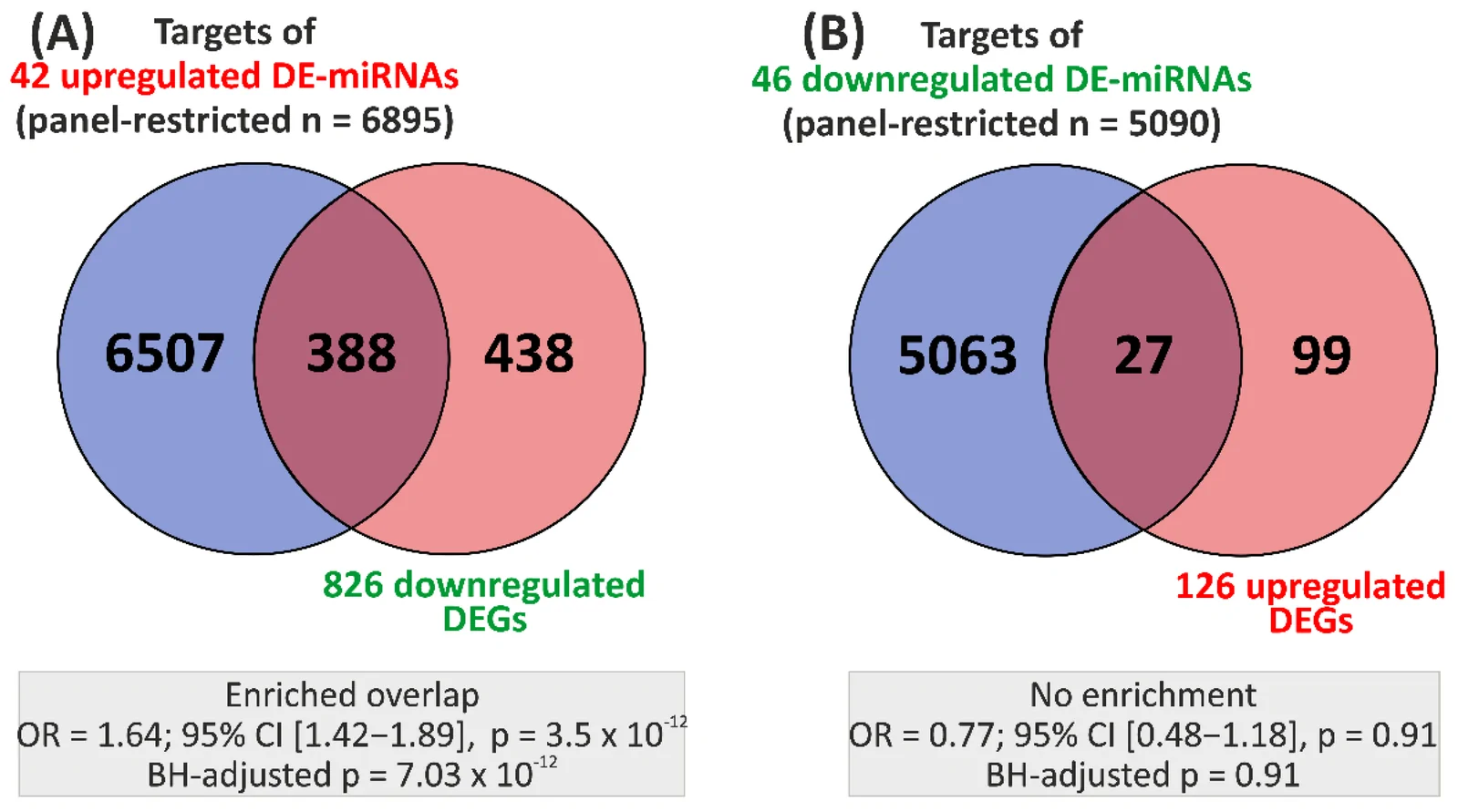
Inverse-direction overlap between experimentally supported DE-miRNA targets and hEDS/HSD DEGs after restriction to the 19,399 genes represented on the Ion AmpliSeq Transcriptome panel. (**A**) Targets of the 42 upregulated DE-miRNAs (*n* = 6895) and the 826 downregulated DEGs shared 388 genes (OR = 1.64; hypergeometric *p* = 3.5 × 10^−12^; BH-adjusted *p* = 7.0 × 10^−12^). (**B**) Targets of the 46 downregulated DE-miRNAs (*n* = 5090) and the 126 upregulated DEGs shared 27 genes (OR = 0.77; *p* = 0.91; BH-adjusted *p* = 0.91).

The 42 upregulated DE-miRNAs had 7260 unique experimentally supported targets, of which 388 overlapped the 826 downregulated DEGs. The 46 downregulated DE-miRNAs had 5332 unique targets, of which 27 overlapped the 126 upregulated DEGs (Figure 4; Table S3). For formal testing, the target lists were restricted to the 19,399 unique gene symbols represented on the Ion AmpliSeq Transcriptome panel, yielding 6895 and 5090 eligible targets, respectively; all 388 and 27 overlapping genes were retained.

The 388-gene overlap was greater than expected by chance (expected overlap, 293.6 genes; odds ratio [OR] = 1.64; 95% CI [1.42–1.89], one-sided hypergeometric *p* = 3.5 × 10^−12^; BH-adjusted *p* = 7.0 × 10^−12^). In contrast, the 27-gene overlap was not enriched above chance (expected overlap, 33.1 genes; OR = 0.77, 95% CI [0.48–1.18]; one-sided hypergeometric *p* = 0.91; BH-adjusted *p* = 0.91). Accordingly, the first overlap provides statistically supported network-level convergence, whereas the second should be interpreted as a descriptive set of candidate interactions. Neither analysis establishes sample-wise miRNA-mRNA correlation or causality.

**Figure 5:**
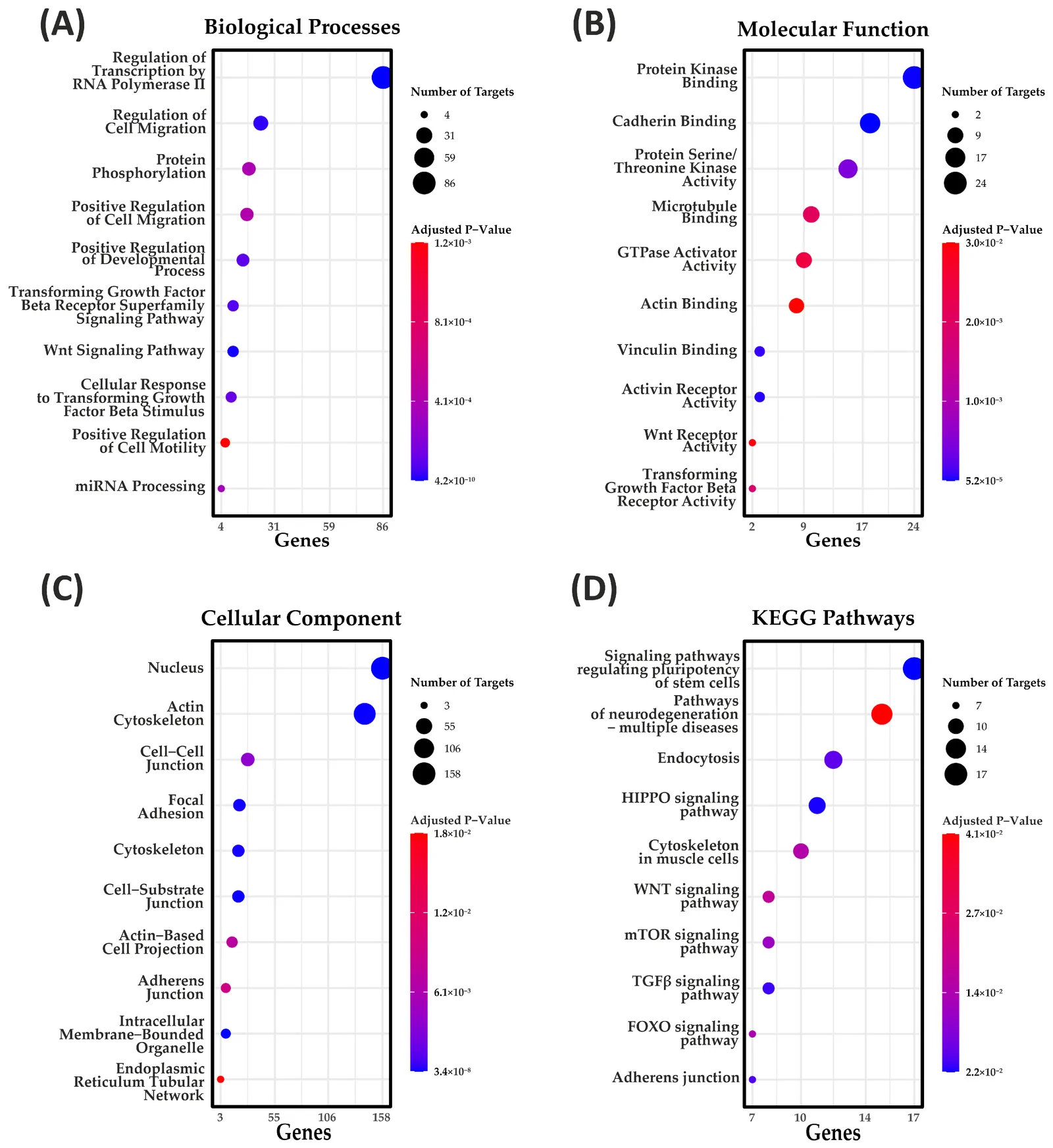
Functional enrichment among the statistically supported 388-gene overlap between targets of the 42 upregulated DE-miRNAs and the 826 downregulated DEGs. Top 10 enriched terms are shown for gene ontology (**A**) biological process, (**B**) molecular function, and (**C**) cellular component categories, and for (**D**) KEGG pathways. Dot size is proportional to the number of target genes assigned to each term (larger dots indicate more genes), and dot color indicates the BH-adjusted *p*-value.

Functional enrichment of the statistically supported 388-gene overlap highlighted Wnt and TGF-β receptor-superfamily signaling, cell migration, cadherin and kinase binding, focal adhesion, actin cytoskeleton, and Hippo/mTOR pathways; key contributors included SMAD1, BMPR2, TGFBR1, WNT2/WNT2B, FZD6, SNAI1, FOS, and FERMT2 (Figure 5; Table S4).

**Figure 6:**
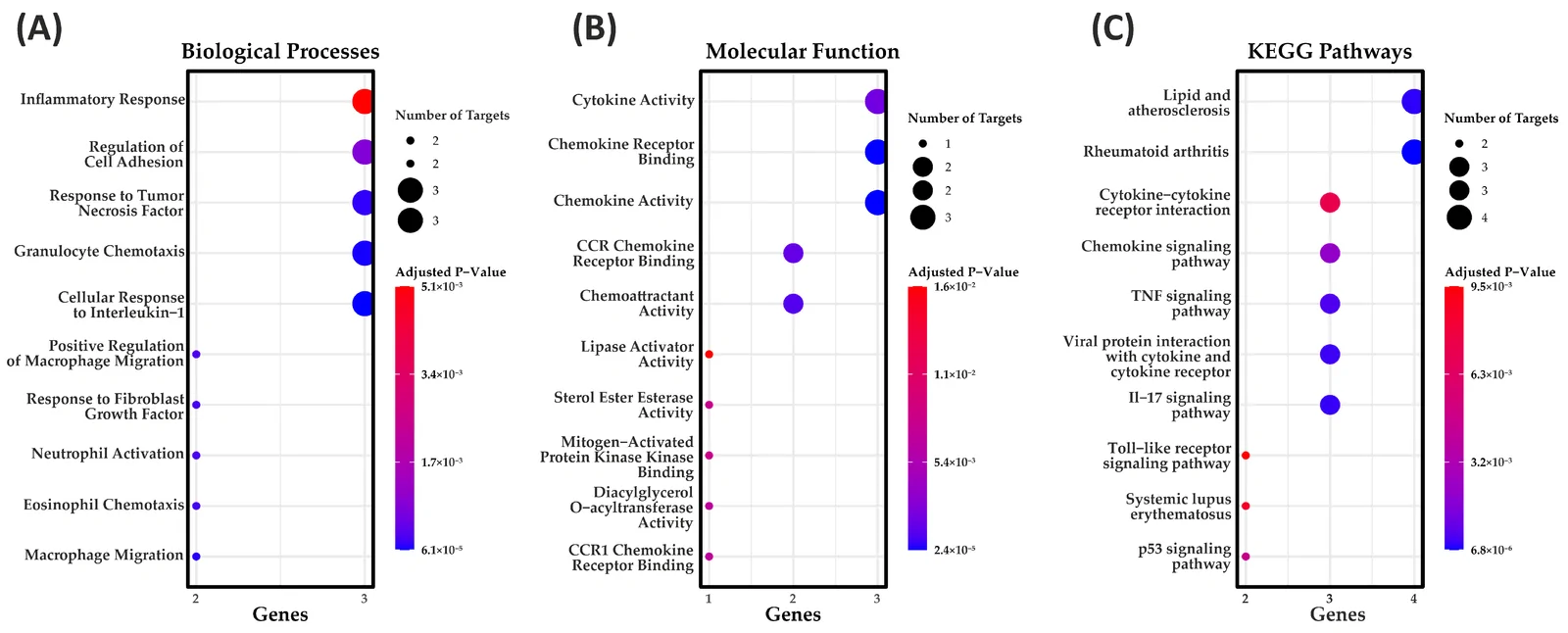
Functional enrichment among the 27-gene overlap between targets of the 46 downregulated DE-miRNAs and the 126 upregulated DEGs, which did not reach statistical significance in the hypergeometric enrichment test (OR = 0.77, 95% CI [0.48–1.18]; *p* = 0.91) and should therefore be interpreted as a descriptive, hypothesis-generating gene set. Top enriched terms are shown for gene ontology (**A**) biological process and (**B**) molecular function categories, and for (**C**) KEGG pathways; no cellular component term reached statistical significance for this gene set. Dot size is proportional to the number of target genes assigned to each term (larger dots indicate more genes), and dot color indicates the BH-adjusted *p*-value.

Within the non-significant 27-gene overlap, functional annotation highlighted a compact chemokine-related subset centered on CXCL8, CCL5, and CCL2, with IL-1/TNF responses and granulocyte/macrophage chemotaxis (Figure 6; Table S4). Because the overlap itself was not greater than expected by chance, these pathway annotations are descriptive and hypothesis-generating rather than evidence of statistically robust network-level convergence. Disease-named KEGG terms such as rheumatoid arthritis and atherosclerosis denote shared cytokine modules, not disease equivalence.

Interaction-level inspection prioritized directionally concordant axes for future testing: miR-141-3p/miR-200c-3p/miR-205-5p-ZEB1, miR-200c-3p-FERMT2, miR-205-5p/miR-26b-5p-CCN2, miR-16-5p-ACVR2A/SMAD1/FZD6, and miR-181a-5p/miR-181b-5p-FOS in the statistically supported ECM/mechanotransduction arm; and miR-31-5p-MMP3, miR-214-3p-CCL5, let-7d-5p-CXCL8, and miR-24-3p-CCL2/LAMB3 as descriptive inflammatory/degradative candidates (Table 1 and Table S3). Evidence strength varies among these interactions, and none has yet been functionally validated in hEDS/HSD fibroblasts.

## 3. Discussion

By integrating miRNome and transcriptome data from matched dermal fibroblast systems, this study identifies a shared post-transcriptional architecture across hEDS and HSD. The direct hEDS versus HSD comparison yielded no statistically significant DE-miRNAs, and the three-group PCA showed extensive overlap between hEDS and HSD samples, whereas both patient groups differed from most CTRL samples. These findings parallel our earlier transcriptomic results [12] and support pooled discovery analysis, but neither a non-significant pairwise comparison nor PCA overlap proves biological identity. A particular strength is the continuity between the two omic layers: miRNome profiling used fibroblast strains from the RNA-seq cohort, reducing inter-cohort variability, while qPCR reproduced selected expression directions in an independent pooled cohort. The UMI-based library strategy further supports technical robustness [34].

The most defensible interpretation is therefore molecular continuity at the level of cultured dermal fibroblasts, not categorical equivalence of all patients, tissues, or disease trajectories. The data strengthen the view that the current clinical boundary may divide overlapping biology, while leaving room for sex-, tissue-, age-, disease-stage-, and phenotype-dependent differences that the present design could not resolve [8].

The analytical architecture is also an important contribution. Rather than interpreting the 88 DE-miRNAs in isolation, the study first assessed the collective biology of experimentally supported targets and then imposed directionality by intersecting targets with oppositely regulated DEGs. Restriction to miRTarBase, miRecords, and TarBase reduced reliance on prediction-only associations [32,35,36,37]. Formal overlap testing then separated the evidentiary strength of the two arms: the 388-gene target/downregulated-DEG overlap was significantly enriched, whereas the 27-gene target/upregulated-DEG overlap was not. Thus, the combined 415 genes provide a prioritized candidate map, but they should not be interpreted as a uniformly supported regulatory network.

Candidate prioritization should account for more than fold change alone. Several of the largest effects, including miR-205-5p, miR-141-3p, and miR-200c-3p, arose from low mean read counts, whereas abundant miRNAs such as miR-16-5p, miR-24-3p, miR-31-5p, and miR-22-3p showed smaller but potentially more robust shifts. A translational ranking should jointly consider abundance, effect size, qPCR concordance, reproducibility in individual samples, target-evidence strength, and pathway relevance. The strong agreement observed in both lower- and higher-count strata argues against concordance being driven only by highly expressed miRNAs; nevertheless, pooled qPCR supports group-level reproducibility rather than subject-level variance or diagnostic performance.

The miR-200/205 family illustrates the biological ambiguity of direction alone. Its coordinated upregulation and inverse overlap with ZEB1, FERMT2, and CCN2 could restrain fibrogenic conversion, disrupt adhesion and matrix assembly, or represent feedback to an already abnormal ECM state [38,39,40,41]. Likewise, miR-181a/b deserve priority because they were upregulated here and, in systemic-sclerosis models, directly repressed NUDT21; miR-181 inhibition attenuated, whereas miR-181 mimics aggravated bleomycin-induced dermal fibrosis [27]. The same miRNA can therefore be compensatory in one network and pathogenic in another.

Several downregulated candidates reinforce this concept. miR-196a-5p combined a large decrease with close sequencing-qPCR agreement; in keloid and systemic-sclerosis fibroblasts, loss of miR-196a has been associated with increased *COL1A1* and *COL3A1* expression [42,43]. Reduced miR-22-3p may likewise release TGF-β-driven activation [44]. These findings do not define hEDS/HSD as conventionally fibrotic; rather, they suggest that matrix synthesis, degradation, adhesion, and stress responses are insufficiently coordinated.

The inverse-overlap analysis provides a disease-focused set of hypotheses. The statistically enriched 388-gene set offers the strongest network-level support: miR-141/200c/205-ZEB1, miR-200c-FERMT2, miR-205/26b-CCN2, miR-16-ACVR2A/SMAD1/FZD6, and miR-181a/b-FOS connect the miRNome to TGF-β/BMP, Wnt/Hippo, focal adhesion, and cytoskeletal nodes central to fibroblast activation and force sensing [45,46,47]. These axes are mechanistically plausible, but their direction and phenotypic consequences must be established by perturbation in hEDS/HSD cells.

The 27-gene inverse overlap requires a more cautious interpretation because it did not exceed chance expectation. Functional annotation within this observed set nevertheless highlighted chemokine-related processes and specific experimentally supported candidates, including miR-31-5p-MMP3, miR-214-3p-CCL5, let-7d-5p-CXCL8, and miR-24-3p-CCL2/LAMB3. These relationships provide testable links between reduced post-transcriptional restraint, matrix degradation, chemokine production, and the degradative-inflammatory loop proposed for hEDS/HSD [12], but they currently represent lower-confidence, hypothesis-generating axes rather than a statistically established inflammatory network. Disease-named KEGG terms such as rheumatoid arthritis or atherosclerosis should likewise be read as shared pathway annotations, not diagnoses or comorbidities.

Comparative connective-tissue disease biology supports this network-based interpretation without implying disease equivalence. In vascular Ehlers–Danlos syndrome, miR-29b manipulation restored selected ECM and stress-response targets in patient fibroblasts [23,24]. In systemic and localized scleroderma, miR-181 and miR-196a link dermal fibroblast programs to in vivo fibrosis and circulating signals [26,27,42,43]. Loeys–Dietz syndrome and pseudoxanthoma elasticum further demonstrate how TGF-β/BMP imbalance, altered myofibroblast behavior, and MMP-driven ECM remodeling can converge across genetically distinct matrix disorders [28,29,30,31].

For personalized medicine, the next step is not a single universal miRNA marker but a prospectively validated multi-analyte panel. Leading candidates should be quantified in individual fibroblast strains and paired plasma, serum, and extracellular-vesicle fractions, then related to clinical domains such as joint instability, pain, skin and vascular features, dysautonomia, gastrointestinal involvement, and inflammatory burden. miR-196a-5p and miR-181b-5p are especially relevant for tissue-to-fluid testing because circulating changes have been reported in scleroderma phenotypes [25,26]. In contrast, circulating miR-16-5p requires stringent hemolysis control because erythrocyte contamination can substantially alter its plasma abundance [48]. Standardized collection, platelet depletion, hemolysis assessment, spike-in controls, sex-inclusive cohorts, and repeated longitudinal sampling will be essential before diagnostic or prognostic claims are justified [34,48].

Therapeutic development should likewise proceed from causal rescue rather than expression direction alone. Candidate axes should first be tested in patient-derived fibroblasts and three-dimensional or organotypic matrix models, using miRNA mimics or inhibitors together with target-engagement and rescue assays. Precedent exists in other fibrotic settings: miR-181 inhibitors reduce dermal fibrosis in mice, exosomal miR-22 limits pulmonary fibrosis in vivo, the miR-29b mimic remlarsen produced pharmacodynamic and histologic effects in human skin, and the lung-targeted miR-29 mimic MRG-229 showed antifibrotic activity in human tissue models and animals [27,44,49,50]. However, broad antifibrotic intervention could be counterproductive in hEDS/HSD, where tissue fragility and defective matrix organization, rather than simple collagen excess, are central. In the absence of a validated organismal model for hEDS/HSD, translational evaluation should prioritize patient-derived systems and in vivo assessment of target engagement, biodistribution, and safety rather than assume that an animal model can reproduce the full disorder.

The study has several strengths that should be considered alongside its limitations. It provides, to our knowledge, the first disease-focused miRNome-wide analysis of hEDS/HSD fibroblasts, preserves continuity with a previously characterized transcriptomic cohort, assesses selected signals in two pooled qPCR settings, and combines experimentally supported targets with direction-aware integration and formal overlap testing. The addition of a main-text table summarizing representative axes, together with the extensive supplementary datasets, provides a transparent resource for independent validation and future candidate selection.

Several limitations constrain interpretation. Primary dermal fibroblasts offer a disease-relevant and experimentally tractable model, but ex vivo expansion is itself a biological perturbation. Culture duration, passage number, serum exposure, confluence, oxygenation, substrate stiffness, and other environmental conditions can reshape fibroblast state and miRNA expression. We minimized differential technical bias by processing all strains in parallel at matched early passages, under identical medium, culture-duration, and confluence conditions. Nevertheless, standardization cannot exclude culture adaptation, selective expansion of fibroblast subpopulations, or attenuation of transient in vivo signals. The observed miRNome should therefore be interpreted as a disease-associated state retained under standardized culture conditions rather than as a direct representation of the native dermal or systemic miRNome.

The study was also cross-sectional, moderate in size, and restricted to adult women. qPCR used pooled material and therefore did not measure interindividual variance. No paired plasma, serum, extracellular-vesicle, or additional tissue samples were available, and miRNA levels could not be related to disease severity, specific clinical domains, treatment exposure, or other in vivo measures. Circadian, seasonal, age-related, and longitudinal variation was not assessed and may be partly obscured by cell expansion. Consequently, the present results are preliminary with respect to native in vivo biology, clinical stratification, and biomarker performance, even though they provide a reproducible group-level and hypothesis-generating framework.

Finally, target databases are biased toward well-studied miRNAs, and their experimental evidence varies in strength, assay type, and cellular context. The 9198-target enrichment is intrinsically broad, inverse overlap between independent omic lists does not establish direct regulation or causality, and only the 388-gene arm was significantly enriched above chance. The 27-gene inflammatory/degradative subset must therefore remain explicitly descriptive until validated at the individual, molecular, and functional levels.

These limitations also define a clear validation sequence: reproduce the candidate pattern in independent individuals; evaluate miRNA-mRNA associations across matched individual fibroblast samples; functionally test the highest-priority miRNA-mRNA axes in patient-derived fibroblasts and assess their causal contribution to matrix and inflammatory phenotypes through rescue experiments; and establish concordance across tissues and circulation in age- and sex-inclusive longitudinal cohorts. Such work could convert the present atlas from an association map into a clinically actionable framework for molecular stratification and mechanism-guided intervention. The principal advance is therefore not merely the addition of a- miRNA catalog, but the construction of a coherent framework that connects our previous cellular and transcriptomic observations to statistically prioritized, experimentally testable mechanisms and translational endpoints.

## 4. Materials and Methods

### 4.1. Patients

The study was conducted in accordance with the Declaration of Helsinki and approved by the local Institutional Review Board of ASST Spedali Civili, Brescia, Italy (registration number NP3500). Twelve adults with hEDS (P1–P12; all female), 12 with HSD (H1–H12; all female), and 24 unrelated healthy controls (C1–C24; all female) provided written informed consent for participation and skin biopsy. Patients were evaluated at the Ehlers–Danlos syndrome and related connective-tissue disorders clinic of Spedali Civili of Brescia and diagnosed according to the 2017 EDS nosology [1]. Controls were selected within the patient age range and screened to exclude features or comorbidities relevant to the hEDS framework. All participants had been recruited for the previous transcriptomic study [12], and skin biopsies were processed according to the published protocol [10].

### 4.2. Cell Culture Conditions

Primary dermal fibroblasts were established from skin biopsies and cultured in Earle’s Modified Eagle Medium supplemented with 2 mM L-glutamine, 10% fetal bovine serum, and 100 μg/mL penicillin and streptomycin (Thermo Fisher Scientific, Rodano, Italy). Cells were maintained at 37 °C in a humidified atmosphere containing 5% CO_2_ and used between passages 3 and 5. At confluence, fibroblasts were harvested with 0.25% trypsin/0.02% EDTA and processed at matched passage numbers.

### 4.3. RNA Purification and miRNome Sequencing

Total RNA, including small RNAs, was isolated from patient and control fibroblasts cultured for 72 h to approximately 80% confluence using the miRNeasy Kit (Qiagen, Milan, Italy). RNA quantity and quality were assessed with a Qubit fluorometer (Thermo Fisher Scientific, Rodano, Italy) and an Agilent 2100 Bioanalyzer (Agilent Technologies, Cernusco sul Naviglio, Italy).

Small-RNA libraries were prepared from 100 ng total RNA from 12 hEDS, 12 HSD, and 24 CTRL cell strains using the QIAseq miRNA Library Kit (Qiagen, Milan, Italy). Unique molecular indexes were incorporated during cDNA synthesis to reduce amplification bias [34]. Library quality and concentration were assessed with the QIAxcel DNA High Sensitivity Kit; libraries were diluted to 4 nM, pooled in groups of six, amplified and enriched with the Ion OneTouch 2/ES system, loaded on Ion 540 chips, and sequenced on an Ion S5 instrument.

Adapters were removed with cutadapt v.5.2 (3′ adapter, AACTGTAGGCACCATCAAT; 5′ adapter, GTTCAGAGTTCTACAGTCCGACGATC). Reads shorter than 17 nucleotides were discarded, and miRNAs were quantified with the miRDeep2 pipeline [51] using mature and hairpin sequences from miRBase v.22.1. miRNAs with a mean count < 20 across all samples were excluded. Differential expression was analyzed with DESeq2 v.1.53.2 [52], and DE-miRNAs were defined by a Benjamini–Hochberg-adjusted *p*-value ≤ 0.05.

### 4.4. qPCR Assessment of Selected DE-miRNAs

Selected DE-miRNAs were assessed by qPCR using independently prepared RNA from patient and control fibroblasts.

First, pooled cDNA from the same fibroblast strains used for miRNome sequencing (12 hEDS, 12 HSD, and 24 CTRL) was analyzed to compare group-level fold changes across platforms. Twenty-five DE-miRNAs (15 upregulated and 10 downregulated; Table S2) were selected to represent both directions of differential expression and to span the observed ranges of fold-change magnitude and mean read count. This design tested miRNome-qPCR concordance across highly and moderately dysregulated miRNAs and across high- and low-abundance transcripts rather than only the most extreme candidates. Distributional normality was assessed with the Shapiro–Wilk test, and concordance between miRNome and qPCR log2(fold-change) values was evaluated with Spearman’s rank correlation in R for all 25 miRNAs and separately for mean read counts <100 and >100. BH correction was applied across the three correlation tests.

Second, an independent pooled cohort was assembled from fibroblast strains in the original RNA-seq study that were not included in miRNome sequencing [12]. RNA from 8 hEDS, 8 HSD, and 16 CTRL strains was pooled within groups, and 20 ng of each pool was reverse-transcribed with the miRCURY LNA RT Kit (Qiagen, Milan, Italy).

qPCR was performed with the miRCURY LNA miRNA SYBR Green PCR Kit and miRCURY LNA assays specific for each selected miRNA (Table S5). SNORD48 (YP00203903), SNORD44 (YP00203902), and SNORD38B (YP00203901) were used for normalization, and relative expression was calculated by the 2^−ΔΔCt^ method using the geometric mean of the reference small RNAs. One-sample t-tests were performed in GraphPad Prism v.8.0 on repeated technical measurements from one pooled biological sample per group; these tests quantify technical reproducibility and do not provide subject-level inference.

### 4.5. Identification of Experimentally Supported miRNA Target Genes and Functional Enrichment Analysis

The multiMiR package v.1.30 [32] was used in R v.4.6.0 to retrieve human miRNA–target interactions supported by experimental evidence.

Queries were restricted to the validated components of miRTarBase [35], miRecords [36], and TarBase [37], excluding prediction-only records. The retained evidence included direct functional assays, such as reporter assays, qRT-PCR, and Western blotting, as well as high-throughput binding and expression studies. Evidence types and support classifications were preserved in Table S3 because they are not equivalent in strength or cell-type relevance.

Target-gene sets were analyzed with Enrichr (https://maayanlab.cloud/Enrichr/, accessed on 22 September 2026) [53] using gene ontology biological process, molecular function, and cellular component categories [54] and the KEGG 2021 Human pathway collection [55]. Enrichment used a two-sided hypergeometric test with BH correction; adjusted *p*-values ≤ 0.05 were considered significant. For graphical prioritization of the large input lists, terms were additionally filtered to require at least three genes, at least 7% representation within the annotation, and a kappa-score threshold of 0.7.

To formally assess whether the inverse-direction overlaps exceeded chance expectation, one-sided hypergeometric tests were performed for the two directional comparisons. The background comprised 19,399 unique gene symbols represented on the Ion AmpliSeq Transcriptome panel used in the prior RNA-seq study [12]. Experimentally supported target lists were restricted to genes in this background, yielding 6895 targets for upregulated DE-miRNAs and 5090 targets for downregulated DE-miRNAs; all 388 and 27 observed overlapping genes, respectively, were retained. For each comparison, the corresponding 2 × 2 contingency table was also analyzed with a two-sided Fisher’s exact test in R to derive the OR and its 95% CI; the resulting *p*-values were numerically consistent with those obtained from the hypergeometric test. *p*-values from the two hypergeometric tests were adjusted using the BH method.

### 4.6. Artificial Intelligence (AI)

During manuscript preparation and revision, generative artificial-intelligence tools (ChatGPT v.5.6, OpenAI; Claude v.5.5, Anthropic) were used to support language editing, literature discovery, and drafting. All sources, analyses, scientific interpretations, and final editorial decisions were critically verified by the authors, who take full responsibility for the accuracy and integrity of the manuscript.

## 5. Conclusions

This study advances hEDS/HSD from a purely phenotypic description toward a testable, multi-layer regulatory framework. Across matched omic datasets, the statistically enriched overlap between targets of upregulated miRNAs and downregulated transcripts defines a robust candidate ECM/mechanotransduction network involving TGF-β/BMP, Wnt/Hippo, focal adhesion, and cytoskeletal programs. The smaller inverse overlap associated with downregulated miRNAs did not exceed chance expectation; nevertheless, it nominates specific experimentally supported inflammatory and matrix-degradative axes, miR-31-MMP3, miR-214-CCL5, let-7d-CXCL8, and miR-24-CCL2/LAMB3, for targeted testing. The data do not establish a diagnostic signature or causal therapy, but they provide a transparent hierarchy of candidates for individual-level validation and functional rescue. Because the findings derive from cultured fibroblasts, pooled qPCR, and a cross-sectional female cohort without paired biofluids or phenotype correlations, their in vivo and biomarker implications remain preliminary. If replicated across individuals, tissues, circulation, and longitudinal clinical trajectories, this framework could support molecular stratification and mechanism-guided intervention. Its most consequential implication is not that hEDS and HSD are proven identical, but that their current clinical boundary may overlie a common fibroblast regulatory continuum while preserving distinct patient-level trajectories.

## Figures and Tables

**Table 1:** Representative experimentally supported miRNA–DEG axes identified by inverse-direction overlap analysis.

Target DEGs	Associated DE-miRNAs	Functional Category
*ZEB1*	miR-141-3p, miR-200c-3p, miR-205-5p	EMT/fibroblast state
*FERMT2*	miR-200c-3p	Focal adhesion/mechanotransduction
*CCN2*	miR-205-5p, miR-26b-5p	ECM/fibrosis marker
*FOS*	miR-181a-5p, miR-181b-5p	AP-1/TGF-β signaling
*SMAD1*	miR-16-5p	TGF-β/BMP signaling
*FZD6*	miR-16-5p	Wnt signaling
*MMP3*	miR-31-5p	ECM proteolysis
*CCL5*	miR-214-3p	Chemokine signaling
*CXCL8*	let-7d-5p	Inflammatory axis
*CCL2*	miR-24-3p	Inflammation
*LAMB3*	miR-24-3p	ECM/inflammation

Rows 1–6 correspond to the statistically enriched overlap between targets of upregulated DE-miRNAs (red) and downregulated DEGs (green); rows 7–11 correspond to the non-enriched overlap between targets of downregulated DE-miRNAs (green) and upregulated DEGs (red) (see Supplementary Tables S1, S3 and S4), and should be interpreted as descriptive candidates. miRNA–target support derives from validated multiMiR resources and does not constitute direct validation in hEDS/HSD fibroblasts.

## Data Availability

Data generated or analyzed in this study are included in the article and its Supplementary Materials. Additional data and materials are available from the corresponding authors on reasonable request and subject to human-research ethics requirements. The miRNome dataset has been deposited in the Gene Expression Omnibus under accession GSE341764.

## Supplementary Materials

The following supporting information can be downloaded at https://www.mdpi.com/article/10.3390/ijms27198628/s1.

## Abbreviations

The following abbreviations are used in this manuscript:

AI	artificial intelligence
BMP	bone morphogenetic protein
BH	Benjamini–Hochberg
BP	biological process
CC	cellular component
cDNA	complementary DNA
CI	confidence interval
CTRL	healthy controls
DEGs	differentially expressed genes
DE-miRNA	differentially expressed microRNA
DNA	deoxyribonucleic acid
ECM	extracellular matrix
EDTA	ethylenediaminetetraacetic acid
FBS	fetal bovine serum
FoxO	forkhead box O
GEO	Gene Expression Omnibus
GO	Gene Ontology
hEDS	hypermobile Ehlers–Danlos syndrome
HSD	hypermobility spectrum disorders
IL-17	interleukin 17
ILK	integrin-linked kinase
IQR	interquartile range
KEGG	Kyoto Encyclopedia of Genes and Genomes
MAPK	mitogen-activated protein kinase
MF	molecular function
miRNA	microRNA
MMP	matrix metalloproteinase
mRNA	messenger RNA
mTOR	mechanistic target of rapamycin
OR	odd ratio
PCA	principal component analysis
qPCR	quantitative polymerase chain reaction
qRT-PCR	quantitative reverse-transcription polymerase chain reaction
RNA	ribonucleic acid
RNA-seq	RNA sequencing
SEM	standard error of the mean
TGF-β	transforming growth factor beta
TNF	tumor necrosis factor

## References

[1] Malfait F., Francomano C., Byers P., Belmont J., Berglund B., Black J., Bloom L., Bowen J.M., Brady A.F., Burrows N.P. (2017). The 2017 International Classification of the Ehlers–Danlos Syndromes. Am. J. Med. Genet. Part C Semin. Med. Genet..

[2] Tinkle B., Castori M., Berglund B., Cohen H., Grahame R., Kazkaz H., Levy H. (2017). Hypermobile Ehlers–Danlos Syndrome (a.k.a. Ehlers–Danlos Syndrome Type III and Ehlers–Danlos Syndrome Hypermobility Type): Clinical Description and Natural History. Am. J. Med. Genet. Part C Semin. Med. Genet..

[3] Castori M., Tinkle B., Levy H., Grahame R., Malfait F., Hakim A. (2017). A Framework for the Classification of Joint Hypermobility and Related Conditions. Am. J. Med. Genet. Part C Semin. Med. Genet..

[4] Demmler J.C., Atkinson M.D., Reinhold E.J., Choy E., Lyons R.A., Brophy S.T. (2019). Diagnosed Prevalence of Ehlers-Danlos Syndrome and Hypermobility Spectrum Disorder in Wales, UK: A National Electronic Cohort Study and Case-Control Comparison. BMJ Open.

[5] Gensemer C., Burks R., Kautz S., Judge D.P., Lavallee M., Norris R.A. (2021). Hypermobile Ehlers-Danlos Syndromes: Complex Phenotypes, Challenging Diagnoses, and Poorly Understood Causes. Dev. Dyn..

[6] Copetti M., Morlino S., Colombi M., Grammatico P., Fontana A., Castori M. (2019). Severity Classes in Adults with Hypermobile Ehlers-Danlos Syndrome/Hypermobility Spectrum Disorders: A Pilot Study of 105 Italian Patients. Rheumatology.

[7] Aubry-Rozier B., Schwitzguebel A., Valerio F., Tanniger J., Paquier C., Berna C., Hügle T., Benaim C. (2021). Are Patients with Hypermobile Ehlers–Danlos Syndrome or Hypermobility Spectrum Disorder so Different?. Rheumatol. Int..

[8] Ritelli M., Chiarelli N., Cinquina V., Vezzoli M., Venturini M., Colombi M. (2024). Looking Back and beyond the 2017 Diagnostic Criteria for Hypermobile Ehlers-Danlos Syndrome: A Retrospective Cross-Sectional Study from an Italian Reference Center. Am. J. Med. Genet. Part A.

[9] Castori M. (2021). Deconstructing and Reconstructing Joint Hypermobility on an Evo-Devo Perspective. Rheumatology.

[10] Zoppi N., Chiarelli N., Binetti S., Ritelli M., Colombi M. (2018). Dermal Fibroblast-to-Myofibroblast Transition Sustained by Avß3 Integrin-ILK-Snail1/Slug Signaling Is a Common Feature for Hypermobile Ehlers-Danlos Syndrome and Hypermobility Spectrum Disorders. Biochim. Biophys. Acta—Mol. Basis Dis..

[11] Chiarelli N., Zoppi N., Venturini M., Capitanio D., Gelfi C., Ritelli M., Colombi M. (2021). Matrix Metalloproteinases Inhibition by Doxycycline Rescues Extracellular Matrix Organization and Partly Reverts Myofibroblast Differentiation in Hypermobile Ehlers-Danlos Syndrome Dermal Fibroblasts: A Potential Therapeutic Target?. Cells.

[12] Ritelli M., Chiarelli N., Cinquina V., Zoppi N., Bertini V., Venturini M., Colombi M. (2022). RNA-Seq of Dermal Fibroblasts from Patients with Hypermobile Ehlers–Danlos Syndrome and Hypermobility Spectrum Disorders Supports Their Categorization as a Single Entity with Involvement of Extracellular Matrix Degrading and Proinflammatory Pathomechanisms. Cells.

[13] Filipowicz W., Bhattacharyya S.N., Sonenberg N. (2008). Mechanisms of Post-Transcriptional Regulation by MicroRNAs: Are the Answers in Sight?. Nat. Rev. Genet..

[14] O’Brien J., Hayder H., Zayed Y., Peng C. (2018). Overview of MicroRNA Biogenesis, Mechanisms of Actions, and Circulation. Front. Endocrinol..

[15] Rutnam Z.J., Wight T.N., Yang B.B. (2013). MiRNAs Regulate Expression and Function of Extracellular Matrix Molecules. Matrix Biol..

[16] Piccinini A.M., Midwood K.S. (2014). Illustrating the Interplay between the Extracellular Matrix and MicroRNAs. Int. J. Exp. Pathol..

[17] Xing X., Rodeo S.A. (2024). Emerging Roles of Non-Coding RNAs in Fibroblast to Myofibroblast Transition and Fibrotic Diseases. Front. Pharmacol..

[18] Mehjabin A., Kabir M., Micolucci L., Akhtar M.M., Mollah A.K.M.M., Islam M.S. (2023). MicroRNA in Fibrotic Disorders: A Potential Target for Future Therapeutics. Front. Biosci..

[19] Botor M., Stępień K.L., Jankowska K., Thlon M. (2026). The Role of Non-Coding RNAs in Connective Tissue Diseases: Diagnostic and Therapeutic Potential of MiRNAs, LncRNAs and CircRNAs. Mol. Diagn. Ther..

[20] Botor M., Auguściak-Duma A., Lesiak M., Sieroń Ł., Dziedzic-Kowalska A., Witecka J., Asman M., Madetko-Talowska A., Bik-Multanowski M., Galicka A. (2023). Analysis of MiRNAs in Osteogenesis Imperfecta Caused by Mutations in COL1A1 and COL1A2: Insights into Molecular Mechanisms and Potential Therapeutic Targets. Pharmaceuticals.

[21] Mercier-Guery A., Millet M., Merle B., Collet C., Bagouet F., Borel O., Sornay-Rendu E., Szulc P., Vignot E., Gensburger D. (2023). Dysregulation of MicroRNAs in Adult Osteogenesis Imperfecta: The MiROI Study. J. Bone Miner. Res..

[22] Abu-Halima M., Kahraman M., Henn D., Rädle-Hurst T., Keller A., Abdul-Khaliq H., Meese E. (2018). Deregulated MicroRNA and MRNA Expression Profiles in the Peripheral Blood of Patients with Marfan Syndrome. J. Transl. Med..

[23] Chiarelli N., Cinquina V., Martini P., Bertini V., Zoppi N., Venturini M., Ritelli M., Colombi M. (2024). Deciphering Disease Signatures and Molecular Targets in Vascular Ehlers-Danlos Syndrome through Transcriptome and MiRNome Sequencing of Dermal Fibroblasts. Biochim. Biophys. Acta—Mol. Basis Dis..

[24] Chiarelli N., Cinquina V., Zoppi N., Bertini V., Maddaluno M., De Leonibus C., Settembre C., Venturini M., Colombi M., Ritelli M. (2024). Integrative Multi-Omics Approach in Vascular Ehlers–Danlos Syndrome: Further Insights into the Disease Mechanisms by Proteomic Analysis of Patient Dermal Fibroblasts. Biomedicines.

[25] Wuttge D.M., Carlsen A.L., Teku G., Steen S.O., Wildt M., Vihinen M., Hesselstrand R., Heegaard N.H.H. (2015). Specific Autoantibody Profiles and Disease Subgroups Correlate with Circulating Micro-RNA in Systemic Sclerosis. Rheumatology.

[26] Makino T., Jinnin M., Etoh M., Yamane K., Kajihara I., Makino K., Ichihara A., Igata T., Sakai K., Fukushima S. (2014). Down-Regulation of MicroRNA-196a in the Sera and Involved Skin of Localized Scleroderma Patients. Eur. J. Dermatol..

[27] Mills T.W., Wu M., Alonso J., Puente H., Charles J., Chen Z., Yoo S.-H., Mayes M.D., Assassi S. (2024). Unraveling the Role of MiR-181 in Skin Fibrosis Pathogenesis by Targeting NUDT21. FASEB J..

[28] Wellbrock J., Sheikhzadeh S., Oliveira-Ferrer L., Stamm H., Hillebrand M., Keyser B., Klokow M., Vohwinkel G., Bonk V., Otto B. (2014). Overexpression of Gremlin-1 in Patients with Loeys-Dietz Syndrome: Implications on Pathophysiology and Early Disease Detection. PLoS ONE.

[29] Pedroza A.J., Dalal A.R., Kim J., Duda M., Tognozzi E., Gilles C., Miller D.C., Fischbein M.P. (2025). Loeys-Dietz Syndrome Subtypes Exhibit Distinct Clinical Behavior and Aortic Cellular Transcriptomic Profiles. JTCVS Open.

[30] Faust I., Donhauser E., Fischer B., Ibold B., Kuhn J., Knabbe C., Hendig D. (2017). Characterization of Dermal Myofibroblast Differentiation in Pseudoxanthoma Elasticum. Exp. Cell Res..

[31] Plümers R., Lindenkamp C., Osterhage M.R., Knabbe C., Hendig D. (2023). Matrix Metalloproteinases Contribute to the Calcification Phenotype in Pseudoxanthoma Elasticum. Biomolecules.

[32] Ru Y., Kechris K.J., Tabakoff B., Hoffman P., Radcliffe R.A., Bowler R., Mahaffey S., Rossi S., Calin G.A., Bemis L. (2014). The MultiMiR R Package and Database: Integration of MicroRNA-Target Interactions along with Their Disease and Drug Associations. Nucleic Acids Res..

[33] Bartel D.P. (2018). Metazoan MicroRNAs. Cell.

[34] Wong R.K.Y., MacMahon M., Woodside J.V., Simpson D.A. (2019). A Comparison of RNA Extraction and Sequencing Protocols for Detection of Small RNAs in Plasma. BMC Genom..

[35] Hsu S.-D., Lin F.-M., Wu W.-Y., Liang C., Huang W.-C., Chan W.-L., Tsai W.-T., Chen G.-Z., Lee C.-J., Chiu C.-M. (2011). MiRTarBase: A Database Curates Experimentally Validated MicroRNA-Target Interactions. Nucleic Acids Res..

[36] Xiao F., Zuo Z., Cai G., Kang S., Gao X., Li T. (2009). MiRecords: An Integrated Resource for MicroRNA-Target Interactions. Nucleic Acids Res..

[37] Vergoulis T., Vlachos I.S., Alexiou P., Georgakilas G., Maragkakis M., Reczko M., Gerangelos S., Koziris N., Dalamagas T., Hatzigeorgiou A.G. (2012). TarBase 6.0: Capturing the Exponential Growth of MiRNA Targets with Experimental Support. Nucleic Acids Res..

[38] Qi J., Liu Y., Hu K., Zhang Y., Wu Y., Zhang X. (2019). MicroRNA-205-5p Regulates Extracellular Matrix Production in Hyperplastic Scars by Targeting Smad2. Exp. Ther. Med..

[39] Qian W., Cai X., Qian Q., Peng W., Yu J., Zhang X., Tian L., Wang C. (2019). LncRNA ZEB1-AS1 Promotes Pulmonary Fibrosis through ZEB1-Mediated Epithelial–Mesenchymal Transition by Competitively Binding MiR-141-3p. Cell Death Dis..

[40] Liu M., Song L., Lai Y., Gao F., Man J. (2024). LncRNA FEZF1-AS1 Promotes Pulmonary Fibrosis via up-Regulating EZH2 and Targeting MiR-200c-3p to Regulate the ZEB1 Pathway. Sci. Rep..

[41] Xie Y., van Daelen M., Park R., Almaraz I., Fontenot L., Rieder F., Ho W., Limketkai B., Shih D.Q., Wei M. (2025). MicroRNA Sequencing of Serum Exosomes Reveals MiR205-5p as an Anti-Fibrogenic Factor Against Intestinal Fibrosis in Crohn’s Disease. Int. J. Mol. Sci..

[42] Kashiyama K., Mitsutake N., Matsuse M., Ogi T., Saenko V.A., Ujifuku K., Utani A., Hirano A., Yamashita S. (2012). MiR-196a Downregulation Increases the Expression of Type I and III Collagens in Keloid Fibroblasts. J. Investig. Dermatol..

[43] Honda N., Jinnin M., Kajihara I., Makino T., Makino K., Masuguchi S., Fukushima S., Okamoto Y., Hasegawa M., Fujimoto M. (2012). TGF-β–Mediated Downregulation of MicroRNA-196a Contributes to the Constitutive Upregulated Type I Collagen Expression in Scleroderma Dermal Fibroblasts. J. Immunol..

[44] Kuse N., Kamio K., Azuma A., Matsuda K., Inomata M., Usuki J., Morinaga A., Tanaka T., Kashiwada T., Atsumi K. (2020). Exosome-Derived MicroRNA-22 Ameliorates Pulmonary Fibrosis by Regulating Fibroblast-to-Myofibroblast Differentiation in Vitro and in Vivo. J. Nippon Med. Sch..

[45] Younesi F.S., Miller A.E., Barker T.H., Rossi F.M.V., Hinz B. (2024). Fibroblast and Myofibroblast Activation in Normal Tissue Repair and Fibrosis. Nat. Rev. Mol. Cell Biol..

[46] Seetharaman S., Etienne-Manneville S. (2020). Cytoskeletal Crosstalk in Cell Migration. Trends Cell Biol..

[47] Malek S., Köster D.V. (2021). The Role of Cell Adhesion and Cytoskeleton Dynamics in the Pathogenesis of the Ehlers-Danlos Syndromes and Hypermobility Spectrum Disorders. Front. Cell Dev. Biol..

[48] Kirschner M.B., Kao S.C., Edelman J.J., Armstrong N.J., Vallely M.P., van Zandwijk N., Reid G. (2011). Haemolysis during Sample Preparation Alters MicroRNA Content of Plasma. PLoS ONE.

[49] Chioccioli M., Roy S., Newell R., Pestano L., Dickinson B., Rigby K., Herazo-Maya J., Jenkins G., Ian S., Saini G. (2022). A Lung Targeted MiR-29 Mimic as a Therapy for Pulmonary Fibrosis. eBioMedicine.

[50] Gallant-Behm C.L., Piper J., Lynch J.M., Seto A.G., Hong S.J., Mustoe T.A., Maari C., Pestano L.A., Dalby C.M., Jackson A.L. (2019). A MicroRNA-29 Mimic (Remlarsen) Represses Extracellular Matrix Expression and Fibroplasia in the Skin. J. Investig. Dermatol..

[51] Friedländer M.R., MacKowiak S.D., Li N., Chen W., Rajewsky N. (2012). MiRDeep2 Accurately Identifies Known and Hundreds of Novel MicroRNA Genes in Seven Animal Clades. Nucleic Acids Res..

[52] Love M.I., Huber W., Anders S. (2014). Moderated Estimation of Fold Change and Dispersion for RNA-Seq Data with DESeq2. Genome Biol..

[53] Kuleshov M.V., Jones M.R., Rouillard A.D., Fernandez N.F., Duan Q., Wang Z., Koplev S., Jenkins S.L., Jagodnik K.M., Lachmann A. (2016). Enrichr: A Comprehensive Gene Set Enrichment Analysis Web Server 2016 Update. Nucleic Acids Res..

[54] Aleksander S.A., Balhoff J., Carbon S., Cherry J.M., Drabkin H.J., Ebert D., Feuermann M., Gaudet P., Harris N.L., Gene Ontology Consortium (2023). The Gene Ontology Knowledgebase in 2023. Genetics.

[55] Kanehisa M., Furumichi M., Sato Y., Kawashima M., Ishiguro-Watanabe M. (2023). KEGG for Taxonomy-Based Analysis of Pathways and Genomes. Nucleic Acids Res..

