# Supplementary material for: Multi-Omic Integration of the miRNome and Transcriptome in Dermal Fibroblasts from Hypermobile Ehlers–Danlos Syndrome and Hypermobility Spectrum Disorders Identifies Convergent Candidate miRNA-mRNA Regulatory Networks Linking Extracellular Matrix Remodeling and Inflammation
Source: Int J Mol Sci. 2026 Sep 26;27(19):8628. doi: 10.3390/ijms27198628 (PMC13665575; doi:10.3390/ijms27198628)
Supplement: Supplementary file 1 [file ijms-27-08628-s001.zip › Supplementary Table S2.pdf]

Supplementary Table S2: Comparison of fold-change values calculated by miRNome analysis and qPCR

miRNA	Mean read count	miRNoma log2(fold change)	qPCR log2(fold change)
hsa-miR-205-5p	2,657	**4,464**	**1,235**
hsa-miR-141-3p	5,995	**2,063**	**1,326**
hsa-miR-200c-3p	2,948	**1,913**	**0,982**
hsa-miR-182-5p	2,743	**1,666**	**1,222**
hsa-miR-142-5p	19,906	**1,393**	**0,512**
hsa-miR-153-3p	3,946	**1,162**	**0,423**
hsa-miR-181b-5p	**117,308**	**1,139**	**1,757**
hsa-miR-181a-5p	**418,128**	**1,082**	**0,846**
hsa-miR-146b-3p	4,774	**1,016**	**0,595**
hsa-miR-154-3p	4,980	**1,001**	**0,350**
hsa-miR-199b-5p	**6559,166**	**0,832**	**1,255**
hsa-miR-146b-5p	**310,539**	**0,763**	**0,650**
hsa-miR-148a-3p	**1676,038**	**0,583**	**0,650**
hsa-miR-93-5p	**1609,251**	**0,453**	**0,441**
hsa-miR-16-5p	**29595,975**	**0,446**	**0,345**
hsa-let-7d-5p	**838,568**	**-0,324**	**-0,265**
hsa-miR-24-3p	**10055,716**	**-0,404**	**-0,456**
hsa-miR-185-5p	**241,032**	**-0,544**	**-0,453**
hsa-miR-22-3p	**2609,656**	**-0,566**	**-0,646**
hsa-miR-31-5p	**4872,383**	**-0,570**	**-0,453**
hsa-miR-330-5p	5,433	**-0,974**	**-0,215**
hsa-miR-214-3p	**167,714**	**-1,008**	**-0,821**
hsa-miR-377-3p	75,940	**-1,045**	**-0,423**
hsa-miR-411-3p	6,091	**-1,180**	**-0,215**
hsa-miR-196a-5p	**195,225**	**-1,681**	**-1,924**

Upregulated miRNAs are shown in red, downregulated miRNA are shown in green.
Read count numbers greater than 100 are in bold.
