# Supplementary material for: Multi-Omic Integration of the miRNome and Transcriptome in Dermal Fibroblasts from Hypermobile Ehlers–Danlos Syndrome and Hypermobility Spectrum Disorders Identifies Convergent Candidate miRNA-mRNA Regulatory Networks Linking Extracellular Matrix Remodeling and Inflammation
Source: Int J Mol Sci. 2026 Sep 26;27(19):8628. doi: 10.3390/ijms27198628 (PMC13665575; doi:10.3390/ijms27198628)
Supplement: Supplementary file 1 [file ijms-27-08628-s001.zip › Supplementary Table S5.pdf]

Supplementary Table S5 : Primer sequences used for qPCR validation of the 25 selected DE-miRNAs.

miRNA ID	Target sequence
hsa-miR-205-5p	UCCUUCAUUCCACCGGAGUCUG
hsa-miR-141-3p	UAACACUGUCUGGUAAAGAUGG
hsa-miR-200c-3p	UAAUACUGCCGGGUAAUGAUGGA
hsa-miR-182-5p	UUUGGCAAUGGUAGAACUCACACU
hsa-miR-142-5p	CAUAAAGUAGAAAGCACUACU
hsa-miR-153-3p	UUGCAUAGUCACAAAAGUGAUC
hsa-miR-181b-5p	AACAUUCAUUGCUGUCGGUGGGU
hsa-miR-146b-3p	GCCCUGUGGACUCAGUUCUGGU
hsa-miR-154-3p	AAUCAUACACGGUUGACCUAUU
hsa-miR-148a-3p	UCAGUGCACUACAGAACUUUGU
hsa-miR-196a-5p	UAGGUAGUUUCAUGUUGUUGGG
hsa-miR-411-3p	UAUGUAACACGGUCCACUAACC
hsa-miR-377-3p	AUCACACAAAGGCAACUUUUGU
hsa-miR-214-3p	ACAGCAGGCACAGACAGGCAGU
hsa-miR-330-5p	UCUCUGGGCCUGUGUCUUAGGC
hsa-miR-16-5p	UAGCAGCACGUAAAUAUUGGCG
hsa-miR-199b-5p	CCCAGUGUUUAGACUAUCUGUUC
hsa-miR-146b-5p	UGAGAACUGAAUUCCAUAGGCUG
hsa-miR-181a-5p	AACAUUCAACGCUGUCGGUGAGU
hsa-miR-93-5p	CAAAGUGCUGUUCGUGCAGGUAG
hsa-miR-24-3p	UGGCUCAGUUCAGCAGGAACAG
hsa-miR-31-5p	AGGCAAGAUGCUGGCAUAGCU
hsa-miR-22-3p	AAGCUGCCAGUUGAAGAACUGU
hsa-miR-185-5p	UGGAGAGAAAGGCAGUUCCUGA
hsa-let-7d-5p	AGAGGUAGUAGGUUGCAUAGUU
